# Towards human distance estimation using a thermal sensor array

**DOI:** 10.1007/s00521-021-06193-2

**Published:** 2021-06-15

**Authors:** Abdallah Naser, Ahmad Lotfi, Joni Zhong

**Affiliations:** 1 Nottingham Trent University, Nottingham, England; 2 The Hong Kong Polytechnic University, Hong Kong, People’s Republic of China

**Keywords:** Distance estimation, Thermal sensor array, Human-centred approach, Artificial neural network, Semantic segmentation, Adaptive system

## Abstract

Human distance estimation is essential in many vital applications, specifically, in human localisation-based systems, such as independent living for older adults applications, and making places safe through preventing the transmission of contagious diseases through social distancing alert systems. Previous approaches to estimate the distance between a reference sensing device and human subject relied on visual or high-resolution thermal cameras. However, regular visual cameras have serious concerns about people’s privacy in indoor environments, and high-resolution thermal cameras are costly. This paper proposes a novel approach to estimate the distance for indoor human-centred applications using a low-resolution thermal sensor array. The proposed system presents a discrete and adaptive sensor placement continuous distance estimators using classification techniques and artificial neural network, respectively. It also proposes a real-time distance-based field of view classification through a novel image-based feature. Besides, the paper proposes a transfer application to the proposed continuous distance estimator to measure human height. The proposed approach is evaluated in different indoor environments, sensor placements with different participants. This paper shows a median overall error of $\pm 0.2$ m in continuous-based estimation and $96.8\%$ achieved-accuracy in discrete distance estimation.

## Introduction

Distance estimation for human monitoring systems is critical in many vital applications. Specifically, human-centred applications require human localisation such as activity of daily living (ADL) recognition and the detection of abnormal human behaviours. Likewise, human distance estimation systems have been extensively used during pandemic periods to prevent the transmission of contagious diseases, such as the coronavirus disease (COVID-19) [23, 34]. Also, through advances in sensor and artificial intelligence techniques, human monitoring systems have become the focal point to cope with long-term care demands of ageing by enabling the older adults to live independently in their own homes [1]. The importance of caring systems for older adults stems from the fact that providing care services to older adults is costly and will increase as the ageing community is increasing [28]. On the other hand, the medical resources for elderly care are becoming scare [7]. Therefore, there is a necessity for human localisation approach to enable existing systems better to cope with complex human behaviour recognition in environments supporting the independent living of older adults.

Typically, human distance measurement systems rely on using a pair of red–green–blue (RGB) cameras [14]. Most of the demonstrations of these works were focusing on their accuracy, sensitivity, and specificity [29]. However, the installation and usage of multiple cameras are an expensive and complicated process. Moreover, it raises more and more serious concerns about users’ privacy in a home environment. Thus, there is a trade-off between performance, privacy, and the cost of sensing approaches for human monitoring applications in the domestic environment.

Following our previous studies [24, 25], this paper proposes a privacy-preserving, non-contact, and low-cost thermal-sensing approach for human distance estimation using a thermal sensor array (TSA). The motivation for using this kind of sensor to estimate human distance is its low-cost sensor compared to regular thermal cameras. Furthermore, the sensor maintains people’s privacy in home-based applications as its output is low-resolution pixel thermal images. In summary, the main contributions of this paper include:

- A novel real-time feature to classify the sensor’s field of view (FoV) into distance-based regions;
- A discrete distance estimation approach to predict human distance in a step of $0.5\,m$;
- A novel continuous distance estimation approach to estimate the distance between the sensor placement and the human location using artificial neural network (ANN);
- A transfer application to predict the human height using the proposed continuous distance estimator;
- Performed robust analysis of the proposed distance estimation approach.

The remaining parts of this paper are organised as follows: in Sect. 2, a summary of the related work regarding human distance estimation is presented. Sect. 3 explains the proposed framework architecture. Experimental results are presented and discussed in Sects. 4 and 5 followed by pertinent conclusions drawn in Sect. 6.

## Related work

Several different solutions have been proposed to estimate the object distance from a camera [10, 36, 38]. However, these techniques usually violate user privacy, especially in home environments.

Based on only the radiation emitted from the human body, TSA is a privacy-preserving sensor. Its construction is typically from a series of connected thermocouples [13] for sensing infrared (IR) radiation. Unlike passive infrared sensor (PIR), TSA’s sensing methodology is based on measuring the total amount of the incident IR flux instead of its change. Therefore, it can detect the stationary target of the FoV’s objects. The TSA has been proposed for passive human positioning in several works reported in [6, 12, 16, 18, 27, 38]. However, none of these works measures the distance between human and sensor placement.

Processing the TSA thermal pictures is similar as image processing approaches [8] with different analytical techniques on individual time intervals (frames) such as support vector machines (SVM) [5, 22], Kalman filtering [19, 37], decision trees [12, 35], adaptive boosting [24, 25], and K-nearest neighbour (KNN) [3, 33]. One of the main technical challenges in human-centred applications using TSA is the temporal disappearance of subjects. This is due to noisy reading, e.g. humans generate noisy heating while moving [24] and external heat sources such as animal pet [26]. Therefore, it is imperative to have a robust background filter to segment the human presence from a noisy background for deployable systems.

The work reported in [2] proposed a ceiling-mounted TSA for counting the number of people up to four. It adopted the SVM classification and motion direction estimation using cross-correlation between the time series of pair pixels. Although their approach did not explicitly discuss the human distance estimation, the proposed processing methodology can be useful for human localisation. The TSA has also been using for activity recognition [15], occupancy detection [4], fall detection [20], and pose detection [9].

Other approaches considered the effects of non-human heat sources acquired by the TSA in human localisation problem such as the work reported in [30]. In this approach, the human shape was considered to filter the non-human presence. However, the human shape varies depending on the TSA placement and sensor to human distance. This raises a serious concern about the adaptability feature of such an approach.

To summarise, the TSA sensors have started to be used due to its low-cost and privacy-preserving. Nevertheless, to the best of our knowledge, an adaptive approach to human distance estimation using the TSA is not reported by other researchers yet.

## Thermal sensing for human distance estimation

**Fig. 1:**
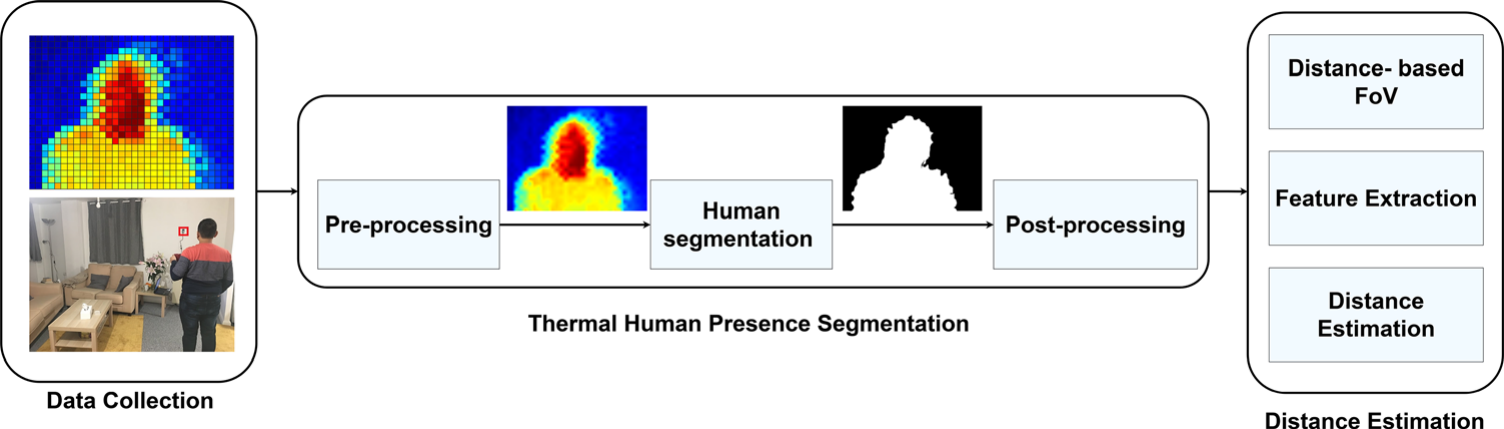
The proposed framework for estimating the distance between the human presence and the thermal sensor array placement after applying a set of techniques, which semantic segment the human presence, followed by a technique to classify the FoV into distance-based regions, and finally output the predicted human distance in the FoV

The sensing approach in the proposed framework is based on the MLX90640 TSA sensor^1^. This sensor is a privacy-preserving sensing approach compared to regular cameras as it produces low-resolution heat-maps. The heat-maps are generated by measuring objects’ temperatures in the sensor’s FoV and displaying them in a $32 \times 24$ matrix. The sampling rate of this sensor can be chosen between $0.5\,Hz$ and $64\,Hz$, and this capability enables us to detect fast movements for human-centred applications.

A schematic diagram of the proposed human estimation approach is shown in Fig. 1. The proposed approach takes into account the characteristics of the TSA, which are different from regular cameras. In contrast with regular cameras which are sensitive to light, TSA is not sensitive to light. Instead, the TSA is sensitive to the environmental radiation compared to the camera, which results in a lot of noise in the TSA images. For example, the edges of the human body in thermal images obtained from TSA are not sharp. The moving body in thermal scenes changes the occupied area’s temperature and surrounding. Therefore, although both the camera-based and TSA-based sensing generate images, their processing techniques are different. In the next sections, a detailed description of the proposed processing framework for TSA will be provided.

### Human presence segmentation

The first stage in the proposed approach consists of three sequential phases (pre-processing, semantic segmentation for the Human presence, and post-processing). The description of these phases is provided as follows.

#### Pre-processing

To enhance the resolution of TSA-based thermal images, an interpolation by 3 factor of the original thermal images is applied. By doing so, the resolution of the obtained turns into $96 \times 72$ instead of its original size $32 \times 24$. Concerning the distance estimation problem versus the TSA characteristics, the minimum captured human temperature varies depending on the sensor’s distance and the human location. Conversely, the maximum human temperature can be determined from the closest point, which is $33\,^\circ$C using the MLX90640 sensor.

**Fig. 2:**
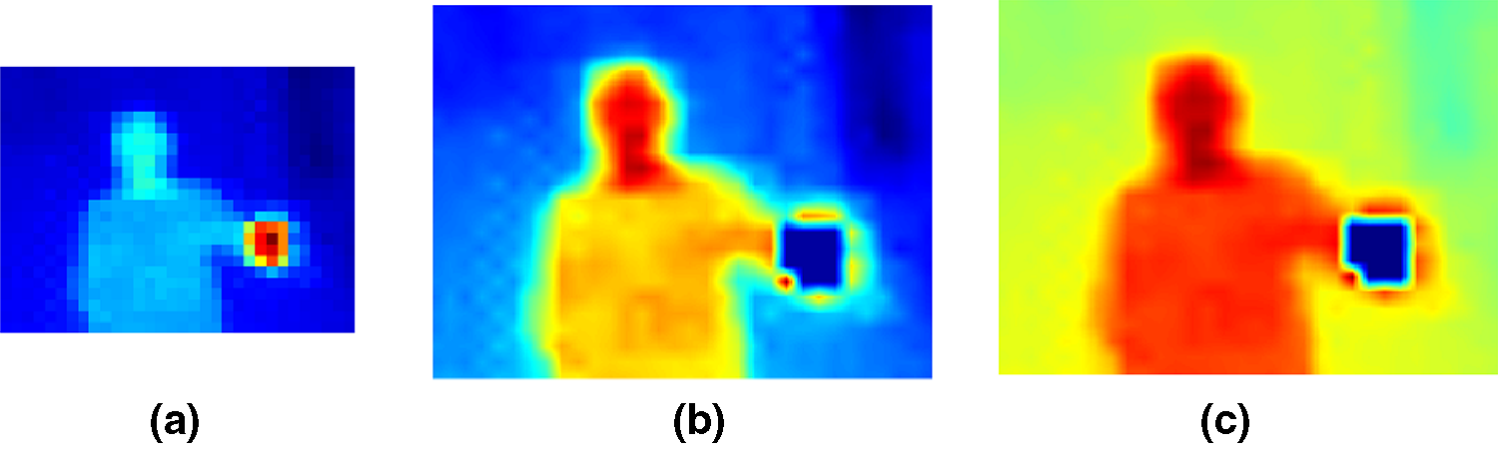
Illustrative results of the pre-processing techniques, **a** the original heat-map of a human holding a cup of coffee, **b** the heat-map after filtering and interpolating the original heat-map, **c** the effect of the faulty filter on the interpolated heat-map

Based on this, any abnormally high temperatures such as a hot kettle can be filtered. On the other hand, it is important to maintain the variance between the minimum and maximum temperatures. So this proposed filter converts the detected high-temperature values to the minimum temperature in the thermal image itself rather than converting the abnormal human high-temperature values to zero. To give an impression, Fig. 2 illustrates the results of applying the pre-processing techniques on TSA’s output. Figure 2a shows the original heat-map acquired while one person is holding a cup of coffee in the sensor’s FoV. Figure 2b shows the result of applying interpolation and the maximum temperature filter. Figure 2c shows a negative example of a wrong, abnormal human temperature filter that converts high-temperature values to zero instead of minimum temperature value in the thermal scene. Although filtering the high-temperature values in the acquired heat-map to zero preserves the human presence in the foreground of the thermal image, it also increases the thermal noise in the background, as well as a loss of visual thermal information (e.g. the heat distribution within the human presence area). As a result, after the pre-processing, the resultant TSA output is an one-channel temperature matrix, and these figures are generated by applying a colour mapping scheme to visualise the TSA output better. Thus, the last step of the pre-processing is exporting the colour mapped matrix into an RGB image to enable the proposed encoder–decoder convolutional neural network to segment human presence as described in the next subsection.

#### Human presence segmentation

From the example provided above, it can be observed that the TSA provides low-resolution images that do not clearly show the edges of captured objects. This raises a serious concern when it comes to locating the human presence at a far human-sensor distance or from a different sensor placement, e.g. room ceiling instead of wall placement. Due to this high intra-class variation in human presence using the TSA, this paper utilised the previous work [24] to link each pixel in the obtained thermal images to either human or background pixel using an encoder–decoder convolutional neural network, which is referred to as semantic segmentation. Furthermore, the object detection techniques [39] differ from semantic segmentation as its algorithms focus on classifying the image regions into a different class rather than on pixel-wise classification.

A network architecture is used, which composes of 23 convolutional layers and two paths called encoder and decoder [31]. The encoder consists of a typical stack of convolutional and max-pooling layers that aims to capture the context of the TSA output while the decoder path is the symmetric expanding part, which uses transported convolutions to output the accurate localisations of the human.

The network optimisation is reported in [17]. The first squared gradients in the optimiser are the mean, *m*, and the second squared gradients are the uncentred variance, *v*. These two gradients are computed as follows:

1 $$\begin{aligned} m_{t}= \,& {} \beta _{1} m_{t-1}+\left( 1-\beta _{1}\right) g_{t} \end{aligned}$$

2 $$\begin{aligned} v_{t}=\, & {} \beta _{2} v_{t-1}+\left( 1-\beta _{2}\right) g_{t}^{2} \end{aligned}$$

where $m_{t}$ is the estimate of the first moment of the gradient, $v_{t}$ is the estimate of the second moment of the gradient, *t* is the index of the training steps. These estimates are biased towards zero, particularly during the initial time steps when the decays rates are small (i.e. $\beta _{1}$ and $\beta _{2}$ are close to 1). The bias-corrected first and second moment estimates are computed as:

3 $$\begin{aligned} {\hat{m}}_{t}= & {} \frac{m_{t}}{1-\beta _{1}^{t}}\end{aligned}$$

4 $$\begin{aligned} {\hat{v}}_{t}=\frac{v_{t}}{1-\beta _{2}^{t}} \end{aligned}$$

Then, the network weight update is calculated as follows:

5 $$\begin{aligned} w_{t}=w_{t-1}-\eta \frac{{\hat{m}}_{t}}{\sqrt{{\hat{v}}_{t}}+\epsilon } \end{aligned}$$

The initial value for $\beta _{1}$ is 0.9, $\beta _{2}$ is 0.999, and $10^{-8}$ for $\epsilon$. The advantage of using the briefly described network is that it can be trained using a small dataset size, and it is suitable for low-resolution images.

#### Post-processing

The primary reason for introducing this post-processing stage is to fill the gaps in the described semantic segmentation technique, with reference to different human conditions and the TSA characteristics. For example, the thickness of the clothes that people wear varies, especially in the home environment. It is possible that the thick clothing could lower the body’s temperature sensed by TSA, resulting in a part of the body being identified as background pixels.

To deal with these human-related issues, an eight-connected filter based on morphological operations [11] is applied to group each object based on its pixel values. In this algorithm, a pixel belongs to the same object if it has the same intensity with its connected horizontal, vertical, or diagonal pixels. Any clustered object with a size less than or equal to 30 pixel is considered as noise and to be removed, e.g. a cup of tea with a similar human temperature. The second remedial image processing technique is to fill in the gaps that may appear in the TSA-based human presence using flood-fill algorithm [21]. As mentioned earlier, the TSA outputs are pre-processed and converted to RGB images to suit the network input. However, the perceived temperature values are lost. Thus, the final stage of this post-processing stage is the recovery of human temperatures using the human presence location found due to applying the semantic segmentation technique and the pre-processed TSA heat-map prior to the RGB conversion.

### Region-based field of view

Based on geometry, it is possible to determine the distance, *D*, between the sensor and an object if the object’s dimension, *O*, is known and the whole object is covered by the sensor’s FoV. That is:

6 $$\begin{aligned} D=\frac{O}{2 \times \tan \left( \frac{F O Y}{2}\right) } \end{aligned}$$

**Fig. 3:**
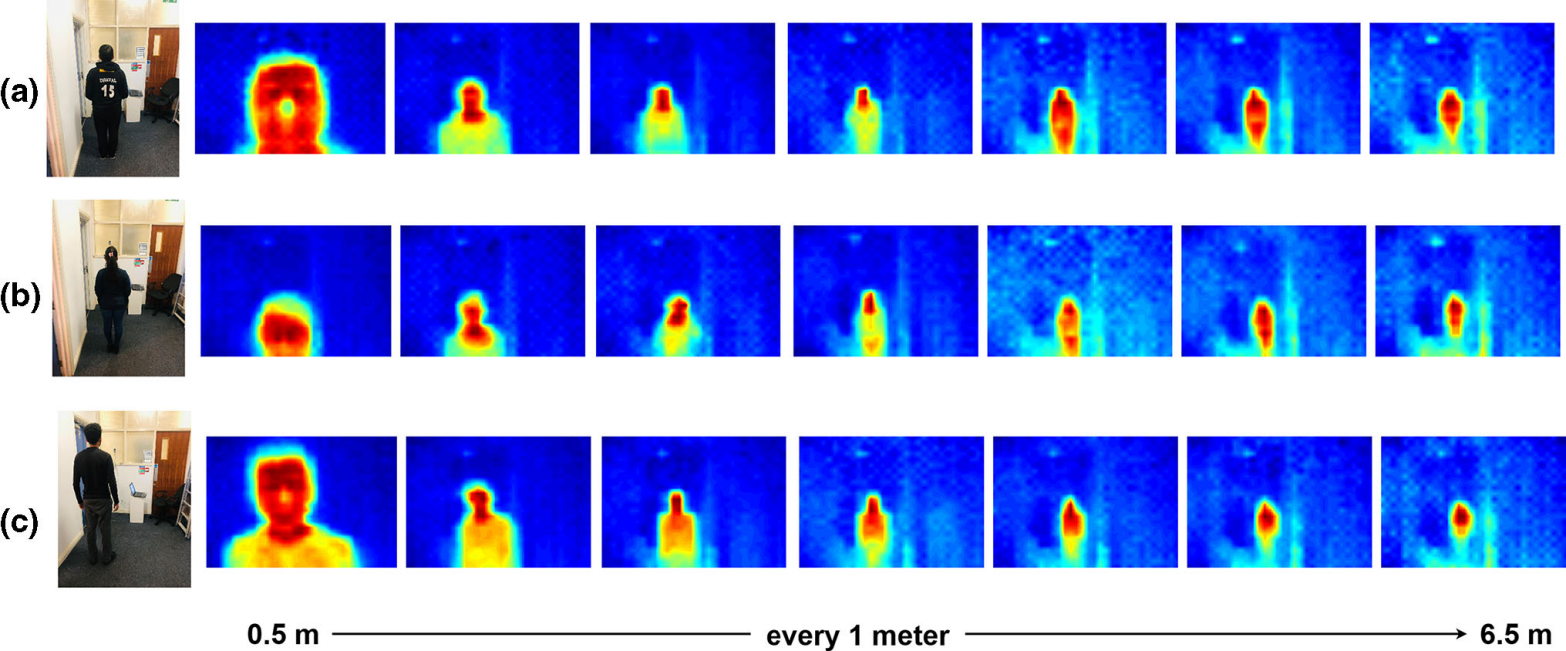
Distance aspect of thermal human presence at distances from 0.5 to 6.5 m in a distance step of 1 m, **a** male participant, **b** short female participant, **c** a relatively tall male participant

However, this geometry does not apply to human-centred sensing applications by TSA as humans vary in body shape in the output images. Figure 3 shows a visualisation of the TSA output used for three participants at distances ranging from 0.5 to 6.5 m with a step of 1 m. From these three illustrative examples, it can be observed at the distance of 0.5 m that the participant in Fig. 3a had his head fully visible while this was not the case for the female participant in Fig. 3b. Continuously for a relatively tall participant, e.g. in Fig. 3c, the head and parts of the upper body are sensed from the same sensor placement. On the other hand, the human body begins to fully emerge in the TSA output at a distance of 3.5 m and beyond. This means the distance for the first few meters is unpredictable using the above geometry, and to predict the distance after 3.5 m, the human dimension is required.

**Fig. 4:**
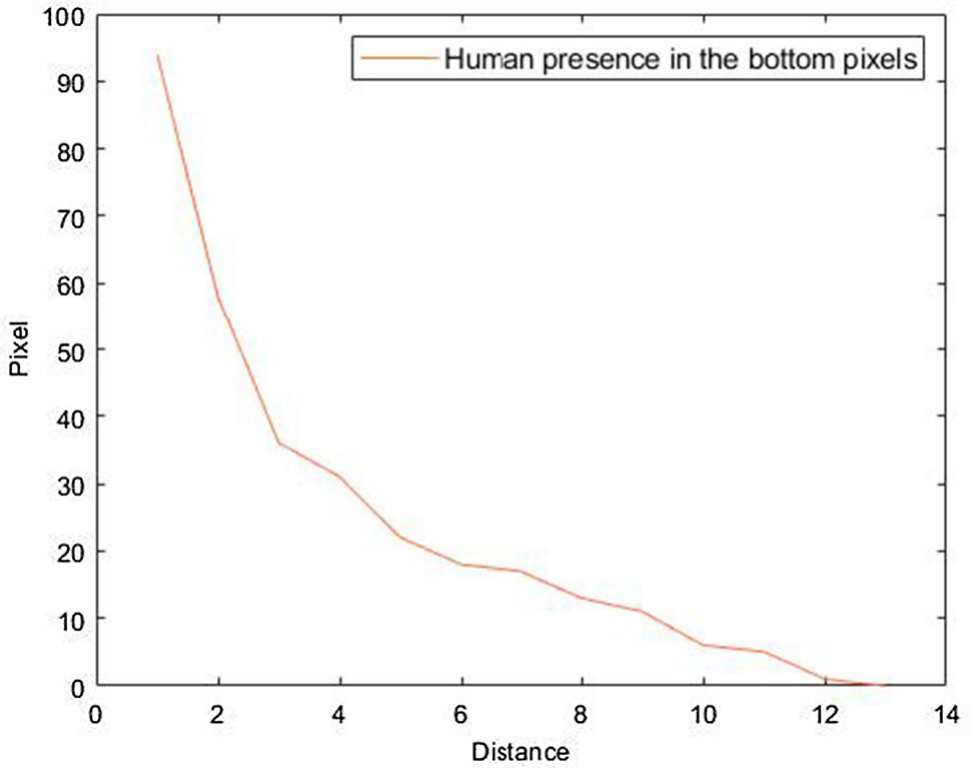
The number of occupied human presence pixels at the bottom of the image versus human to sensor distance

The human distance in the TSA field of view should be carefully estimated. To achieve this, a novel image-based feature to solve this problem is proposed. This feature is based on the observation that human presence diminishes in the bottom rows of the thermal image as the human goes further from the sensor location. Figure 4 shows an example of the bottom image rows of a human moving from a close point to a point far away to the location of the sensor. It can be seen that the number of human pixels at the bottom rows of the thermal image decreases as the distance between the sensor and the human increases. Based on this, the sensor’s FoV can be classified into distance-based regions, e.g. near, middle, and far regions depending on the human presence’s location using the number of occupied human pixels in the bottom rows of the thermal image. Hence, this feature’s simplicity would allow real-time applications to quickly obtain the human location and reduce the processing time to compute the exact human distance estimate as described in the next section.

The human presence mask, which is a binary mask that corresponds to the class (human or background) of each pixel in the obtained thermal image generated by the proposed encoder–decoder convolutional neural network, is used to count the occupied human pixels in the bottom rows of the thermal scene. In other words, count the last nonzero values in the mask mentioned earlier. This feature is then used to train a classification model to predict the region of the human location in the FoV as described in Sect. 4.

### Human distance measurement

In this section, the exact estimate of human distance will be computed after finding the region of human presence in the sensor’s FoV as described in the previous section. Reducing the number of actual distance classes by categorising the FoV into regions results in the reduction in the processing time and increase in the proposed estimation system’s performance. Thus, this section provides a detailed description of the extracted features used to train and test the proposed estimation models which find the human presence region of each human in the sensor’s FoV.

#### Feature extraction

**Fig. 5:**
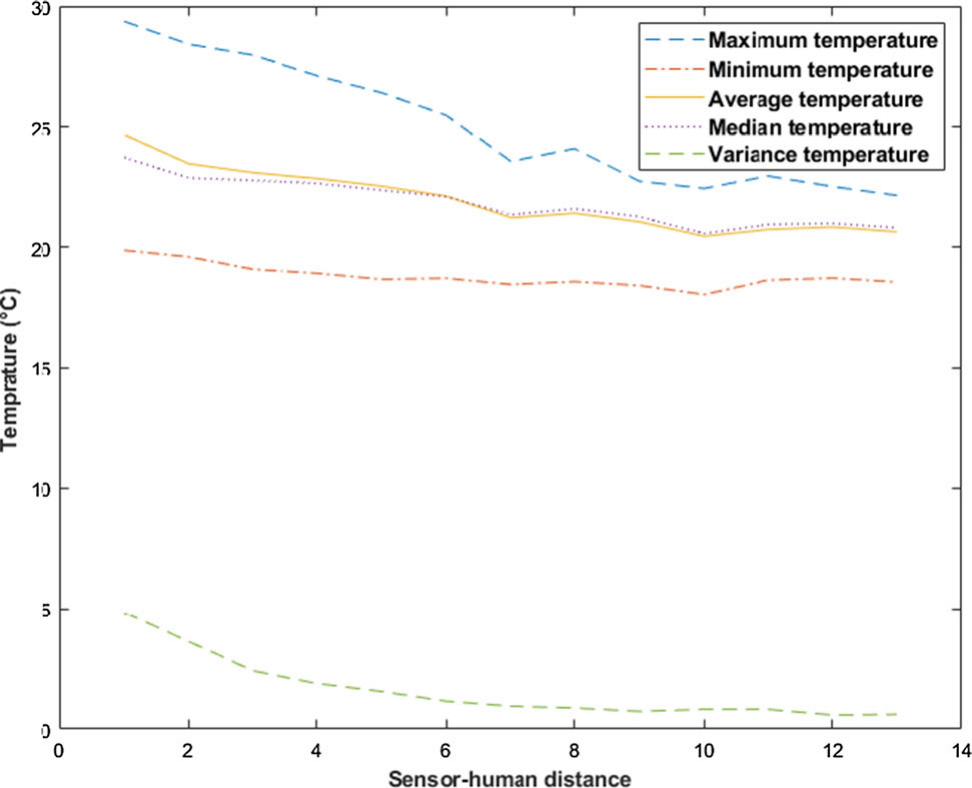
The effect of the distance on the acquired human temperature using the TSA

A number of TSA-based features have been extracted to predict the exact human location to measure the distance between the sensor and a human. Figure 5 shows an evaluative example of the effect of distance on a human temperature captured by TSA on the segmented human heat-map. Specifically, the minimum, maximum, average, mean, median, and variance temperature of human are present from 0.5 to 6.5 m with a distance step of 0.5 m. It can be seen the overall trend human temperature decreases with the increase in the sensor to human distance. To further evaluate the image, the entropy is extracted for each segmented human heat-map histogram using the following equation:

7 $$\begin{aligned} H(X)= \,& {} -\sum _{i=1}^{n} P\left( x_{i}\right) \log P\left( x_{i}\right) \nonumber \\ where \quad n= \,& {} histogram \ bins \end{aligned}$$

In addition to temperature-based features, human presence size was also considered to feed the human distance estimation model. Hence, it has been previously shown that there is an inverse relationship between distance and the size of human existence.

#### Sensor-to-human distance estimation

The first proposed human distance estimation technique is a regression to map between the extracted features *x* and the sensor-to-human distance using artificial neural network. In particular, multilayer perceptron (MLP) artificial neural network with one input layer, one hidden layer with sigmoid neurons, and one output layer is used. The weight updating $\Delta w_{{jk}}$ can be written as:

8 $$\begin{aligned} \Delta w_{\mathrm {jk}}(\mathrm {p})=\eta \times \mathrm {y}_{\mathrm {j}}(\mathrm {p}) \times \delta _{\mathrm {k}}(\mathrm {p}) \end{aligned}$$

where *p* refers to the number of iterations used to propagate the error signal from the output layer to the hidden layer. The gradient error $\delta _{\mathrm {k}}(\mathrm {p})$ in the output layer is determined from the derived activation function multiplied by the error in the output layer neuron. Hence, $\eta$ refers to the learning rate. In this paper, since the estimation is well behaved, the network is trained using the Levenberg–Marquart backpropagation algorithm [32]. This algorithm tries to minimise the sum of the squares of deviations $S({\beta })$ of a set of pair *n* $\left( x_{i}, {\hat{y}}_{i}\right)$ of input heat-maps *x* and the sensor-human distance ŷ by finding the parameters $\beta$ of the model output $f(x, \varvec{\beta })$.

9 $$\begin{aligned}&{\hat{\varvec{\beta }}} \in {\text {argmin}}_{\beta } S(\varvec{\beta }) \nonumber \\&\quad \equiv {\text {argmin}}_{\varvec{\beta }} \sum _{i=1}^{n}\left[ {\hat{y}}_{i}-f\left( x_{i}, \varvec{\beta }\right) \right] ^{2} \end{aligned}$$

The detection of the mean square error of the validation dataset leads to terminate the training process. In a real-life scenario, there is an infinite number of distance classes as one human could be at any distance in the sensor’s FoV. Thus, the aim to utilise this ANN architecture is to find a continuous-based sensor-human distance estimate. However, a discrete-based human distance estimation using classification approach is also performed to evaluate the extracted TSA-based features’ performance by having a specified number of classes for every 0.5 m up to 6.5 m, making a total of 13 classes.

## Experiments

To evaluate the performance of the proposed framework of human distance estimation, experiments were performed using two different configurations of the sensor’s placements. They were also evaluated from three different indoor environments in the summer and winter seasons of the UK. The reason for considering different seasons and different indoor environments is that during the winter season, the indoor heating systems in the UK usually operate. In the summer months, neither heating nor cooling is used. These evaluations ensure a high generalisation ability for the proposed estimation system as the TSA sensor is sensitive to ambient temperature.

**Fig. 6:**
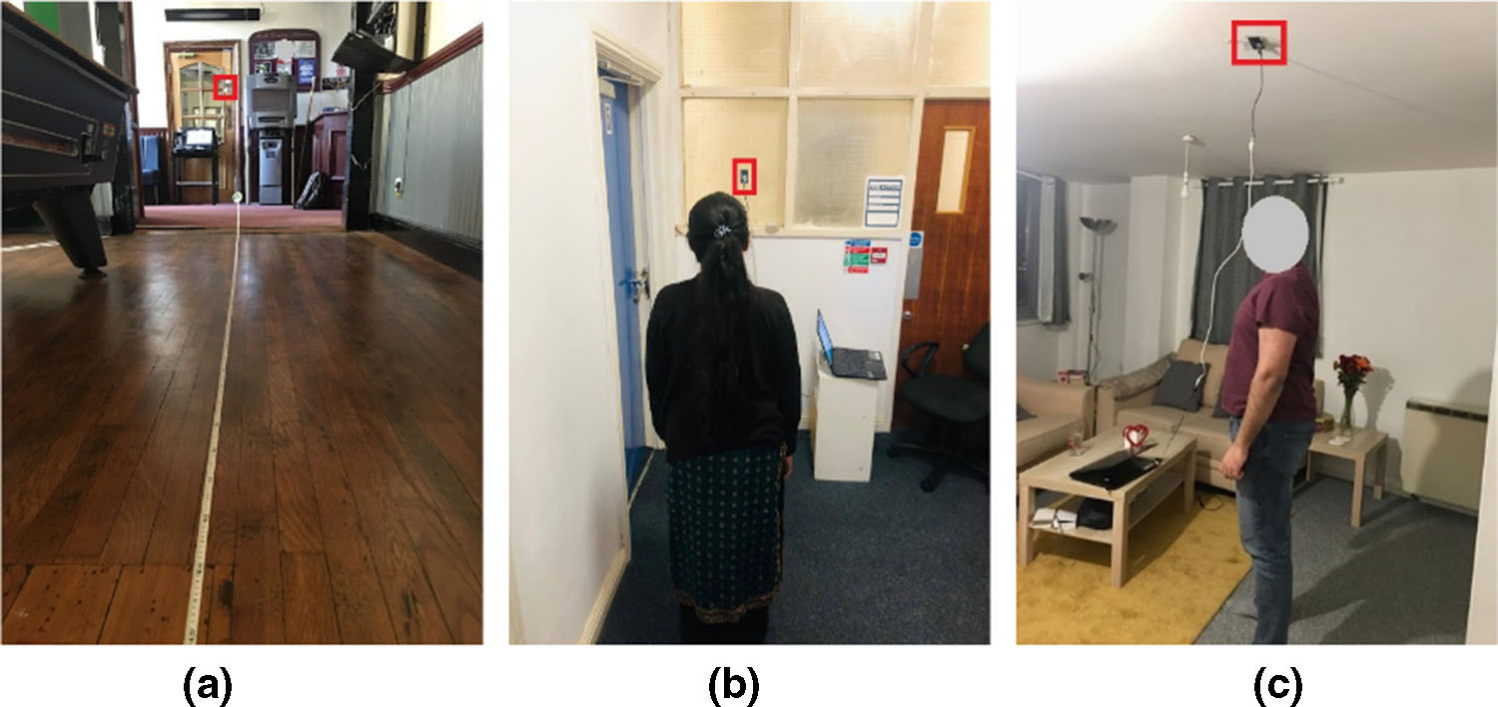
Data collection stages from three different indoor environments, **a** the sensor is placed on the wall to assess the performance of the proposed sensor-human distance methodology, **b** the sensor is also placed on the wall, **c** the sensor is on the ceiling to assess the generalisation of the proposed methodology

In the first data collection configuration, the sensor was placed in a vertical position with a height of 1.57 m from the ground as shown in Fig. 6a and b. A total number of 703 thermal images were collected for six different human participants at distances from 0.5 to 6.5 m every 0.5 m. During this data collection stage, participants were asked to stand on these 13 different distance classes to avoid the over-fitting problem during the algorithms’ learning and testing phases.

The second data collection configuration aims to assess the proposed distance estimation system’s adaptability versus sensor placement and human data bias. At this stage, the low-resolution thermal scenes of two new participants (male and female) were acquired from an overhead sensor placement, as shown in Fig. 6c. The size of this dataset is 90. In total, 793 thermal scenes collected to conclude the results of this paper.

### Region-based FoV experimental results

**Fig. 7:**
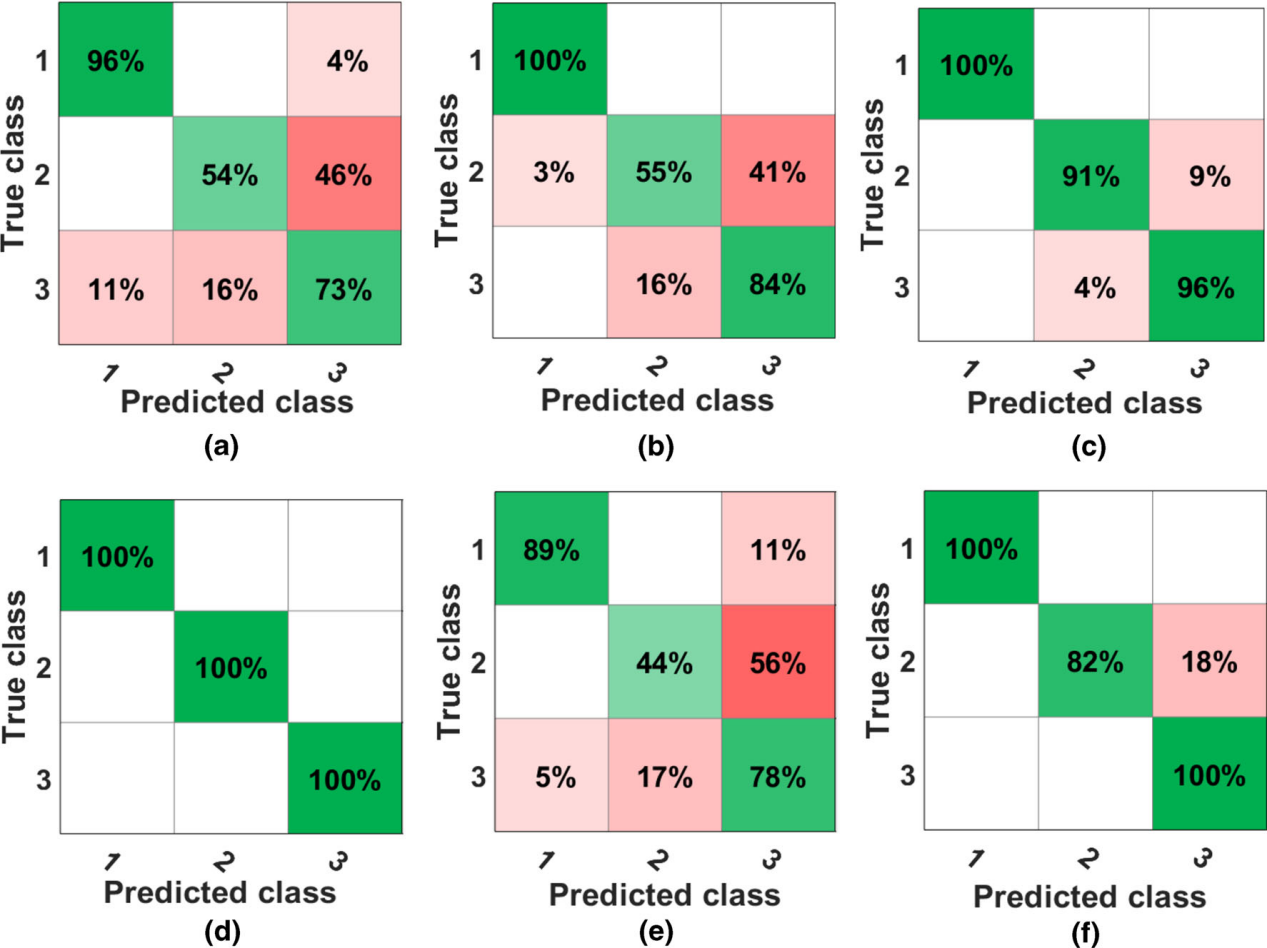
A visualisation of the participant-focused performance of the proposed image-based feature to classify the sensor’s FoV into distance-based regions, where **a**,**c**, **d**, and **f** are confusion matrices for different male participants while **b** and **e** are for female participants

The first experiment examined the proposed image-based feature to categorise the sensor’s FOV into three regions based on the sensor to human distance. The first defined region is from $0\,m$ to $2.5\,m$, the second region ranges from 3 to 4.5 m, and the last one is between 5 and 6.5 m. The used dataset was partitioned into fivefold to protect against over-fitting, and the best overall achieved accuracy was $76.8\%$ using decision trees. Further, focused experiments with same data partition configuration were conducted on each user’s data; Fig. 7 illustrates the proposed image-based feature’s performance on six different human participants. The confusion matrices shown in Fig. 7b and e are for female participants while Fig. 7a, c, d, and f for male participants.

It can be noted from these figures that the proposed feature works relatively better for male participants than female, with an overall accuracy of $91\%$ while for female participants, the accuracy was $74\%$. This observation does not necessarily imply that the heat signature differs based on human gender, but perhaps females tend to wear heavier clothing compared to males, and this reduces the temperature perceived by TSA. On the other hand, females are generally smaller in size than males, which means that their heat signature size will be smaller than that of the males.

### Human distance estimation experimental results

The first experiment is a continuous estimation of human distance using ANN from a vertical sensor placement described in Sect. 3.3.2. In this experiment, the collected dataset was divided into two subsets. The first subset is the thermal data obtained at decimal distances (0.5, 1.5, 2.5, 3.5, 4.5, and 6.5 m). This subset is used to train the proposed neural network to predict the sensor-human distance using the extracted feature vectors described in Sect. 3.3.1 as the network input and the corresponding distances as the output. This network is then tested with completely unseen data to predict the sensor-human distance. The data are from the second subset at integer distances (1, 2, 3, 4, 5, and 6 m). The median overall error in predicting the distances was $\pm 0.2$ m. Hence, since the trained network’s output is a continuous distance value (not a labelled class), this approach is called a continuous-based human distance estimation.

**Table 1:** A comparison of different classification algorithms to classify the sensor to human distance with 10 cross-validation folds

Classification algorithm	Accuracy (%)
Naive Bayes	63.3%
Tree	83.4%
Enesmble - Bagged Trees	90.8%
Kernel Naive Bayes	91.6%
KNN	96.5%
Cubic SVM	96.8%

The same dataset is then used with 13 defined class labels (0.5, 1, 1.5,..., $6.5\,m$) for all data participants obtained from vertical sensor position. At this experiment, various classification algorithms were used to evaluate the performance of the proposed features. The dataset is divided into the training and testing stages using cross-validation with tenfold. Table 1 shows the performance of these classification algorithms. The best-achieved accuracy was $96.8\%$ using Cubic SVM.

## Robust analysis

The robust analysis contains two main experiments. The first experiment evaluates the adaptability and performance of the proposed image-based feature of a distance-based FoV with a different number of regions. In this experiment, two regions were identified instead of the three suggested in Sect. 4.1. The first defined region ranges from 0 to 3 m, and the second region is from $3.5\,m$ to $6\,m$. Reducing the number of defined FoV regions increases the overall accuracy from 76.8 to $95.4\%$. This increase in performance underlines the robustness of the proposed real-time human localisation feature in terms of the FoV region occupied. Besides, it shows low inter-class variation within the second region between $3\,m$ and $4.5\,m$ with the other two defined regions in the previously defined three regions. Thus, the performance was lower prior merging of the second region.

**Fig. 8:**
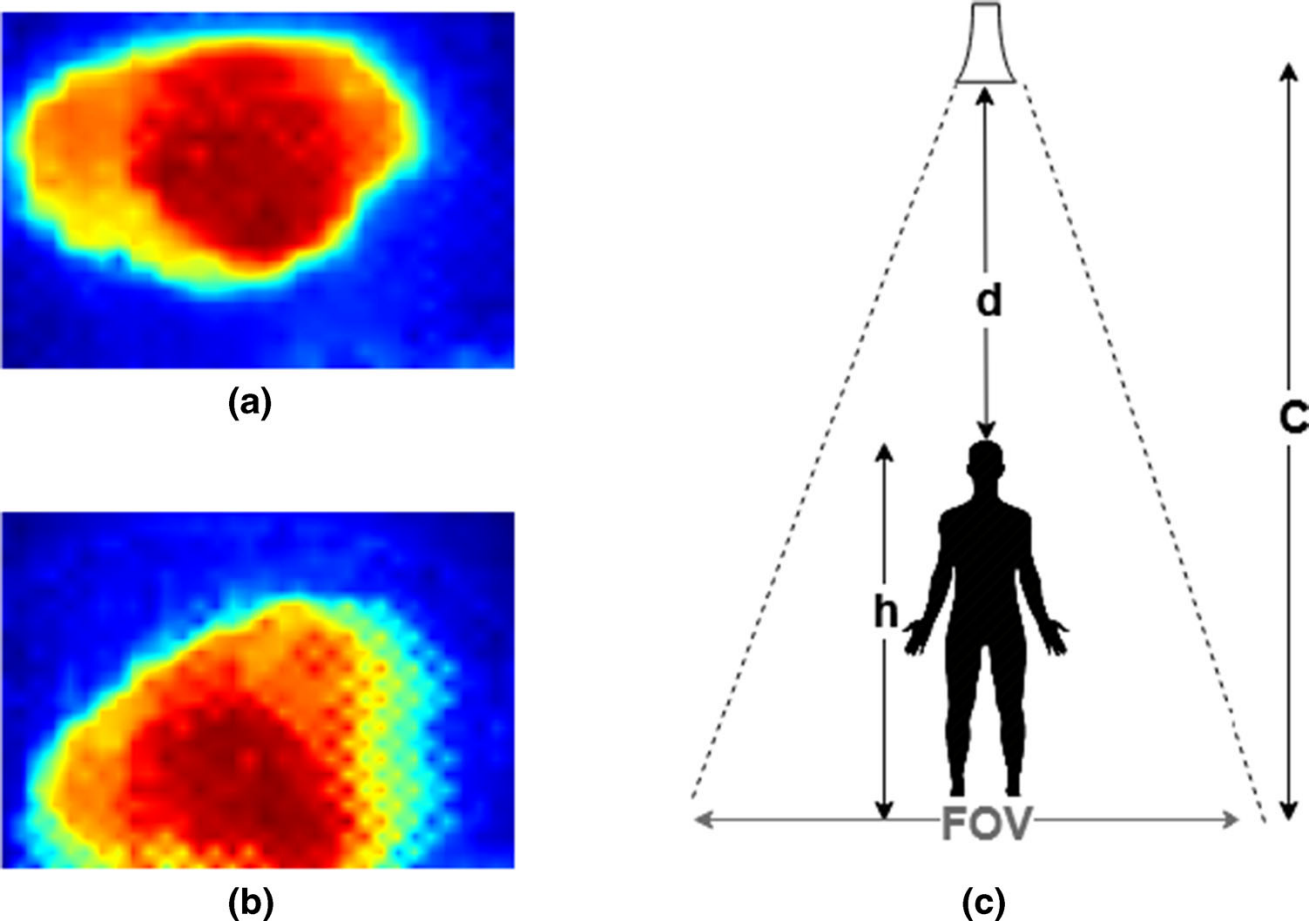
An overhead sensor placement, **a** the overhead image of a fixed-moving human presence, **b** the impact of movement on the thermal human presence, **c** a transfer application of the proposed distance estimator to predict the human height

In the second experiment, the proposed ANN’s generalisation ability to map between the extracted TSA features and the sensor to human distance was assessed. This was achieved through testing the ANN, which is already trained using data obtained from the vertical sensor placement, with completely unseen data obtained from the overhead sensor position and new human participants. The median error in predicting the male participant’s distance was $\pm 0.07$ m and $\pm 0.66$ m for a female participant. Hence, during the data collection phase, the female participant was wearing a headscarf, which reduced her acquired head temperature. Further experiments were performed on the collected data to analyse the impact of the thermal image quality on the performance of the continuous human distance estimator. The fixed human presence had a predictive error of $\pm 0.01$ m while moving human decreases the robustness of the extracted features, resulting in a lower rate of prediction. Figure 8a shows a stable human presence from a sensor placed on the ceiling of the room, and Fig. 8b shows the effects of human movements on the acquired thermal human presence of the same human participant. Importantly, the proposed approach for the estimation of human distance can be transferred to extract human physiological features such as the human height. Given a user case scenario of overhead sensor placement, as shown in Fig. 8c, it is then possible to estimate human height *h* if room ceiling height *c* is known using the following simple geometry:

10 $$\begin{aligned} h=c-d, \quad \text {where}\, d \,\text {is the predicted sensor to human distance} \end{aligned}$$

The robust analyses concluded that the proposed human distance estimation using TSA has high generalisation ability towards operating with different experimental configurations. Besides, the proposed transfer application to measure the human height demonstrates the important impact of the proposed distance estimators on other human-centred applications.

## Conclusion

This paper proposes a privacy-preserving, low-cost, and passive human distance estimation approach based on the thermal sensor array and a tailored image processing framework. The proposed approach has been used for discrete and continuous distance estimation using classification and artificial neural network, respectively, with data collected from different domestic environments. The high intra-class variation in the human shape and heat noises has also been considered through utilising a robust human segmentation technique based on encoder–decoder convolutional neural network, which enables the proposed distance estimator to operate from adaptive sensor placement. Besides, a transfer application using the proposed distance estimator is introduced to extract a human physiological feature (human height).

It can be concluded from the results obtained that the use of TSA, in combination with appropriate processing techniques, could be an approach for human-centred indoor applications. Future work could be undertaken to utilise the proposed approach to measure the physical distance between humans and assess TSA’s use in profiling older adults in smart home solutions.

## Footnotes

1 The sensor details can be obtained from Melexis website: https://www.melexis.com/en/product/MLX90640/
